# Surgical excision of vaginal cysts presenting as pelvic organ prolapse: a case series

**DOI:** 10.11604/pamj.2022.42.10.33537

**Published:** 2022-05-06

**Authors:** Sofia Tsiapakidou, Iakovos Theodoulidis, Grigoris Grimbizis, Themistoklis Mikos

**Affiliations:** 1 1^st^ Department of Obstetrics and Gynecology, Aristotle University of Thessaloniki, Papageorgiou General Hospital, Thessaloniki, Greece

**Keywords:** Vaginal cysts, vaginal surgery, pelvic organ prolapse, excision technique, surgical management

## Abstract

Vaginal cysts are rare, benign, predominantly cystic lesions of the anterior vaginal wall, with a prevalence of 1 in 200 women. Large vaginal cysts can occasionally present as symptomatic genital prolapse; these cases may be challenging to diagnose due to their rare clinical appearance. In symptomatic large vaginal cysts, surgical excision via vaginal approach is the recommended management with good anatomical results and patient satisfaction. The series of three consecutive adult women were referred for bothersome bulging prolapse. They were found to have a sizeable vaginal cyst at the anterior wall, associated with other symptoms. All patients (mean age 37±8.5 years) underwent total trans-vaginal surgical excision of the lesion. They were followed up in the out-patient department at six weeks and six months with no recurrences mentioned. Vaginal cysts are usually solitary, small, and asymptomatic; however, they can increase in size, easily mimic other pathologies, and are misdiagnosed as cystocele. Therefore, complete surgical vaginal excision of the symptomatic vaginal lesion is feasible and constitutes a good management option.

## Introduction

Vaginal cysts are rare, benign, mostly cystic lesions of the anterior vaginal wall, with a prevalence of 1 in 200 women. Vaginal cysts are often asymptomatic, with dimensions less than 2cm, and present as an incidental finding on a gynecological examination. Therefore, they are usually not reported, and their real incidence may be underestimated. Vaginal cysts are more commonly present in the third and fourth decades of life [1]. Regarding the embryological descent and the differential diagnosis, the most common lesions are the Müllerian cysts followed by the epidermal inclusion cysts, the Gartner´s cysts, the Bartholin's gland cysts, urethral diverticulum, and other types of growths of urethral and paraurethral origin [2]. The Müllerian cysts are developed at the 12^th^ embryological week when the squamous epithelium of the Müllerian ducts is replaced by the mucinous epithelium. The epidermal inclusion cysts are formed after vaginal surgical procedures (i.e. an episiotomy) when the local epithelium is buried; they are the most common vaginal cysts of no embryological origin. Gartner´s cysts are derived from the Wolffian duct remnants after the 8^th^ embryological week, during the development of the vaginal wall [3,4].

Usually, the small vaginal cysts are asymptomatic, but larger lesions may present with mild discomfort at the genital area, feeling of vaginal fullness, dyspareunia, lower urinary tract symptoms, and urinary incontinence. A general practitioner, a midwife, and a general gynecologist may only rarely evaluate a cystic formation of the anterior vaginal wall. Uncommonly, these lesions may be misdiagnosed as prolapse [5]. During the physical examination, all vaginal cystic lesions should be assessed and described for their location, size, mobility, consistency, tenderness, and progression. For further investigation, imaging using ultrasonography - especially perineal ultrasound - and Magnetic resonance imaging (MRI) may help identify and describe in detail the cyst´s characteristics and its relationships with the surrounding tissues and organs [6]. Small, asymptomatic vaginal cysts require no treatment and warrant a regular follow-up, as they may rarely increase in size due to mucus production. Large, symptomatic cysts usually require surgical treatment. Surgical excision of the vaginal cyst via vaginal approach is the common management approach, with low recurrence rates [7]. Drainage is no longer advised due to infection and recurrence risks [8]. Pathologic confirmation is recommendable to exclude malignancy as they have been reported cases of malignant transformation [9]. This article aims to present a case of a series of large, symptomatic vaginal cysts mimicking anterior vaginal wall prolapse, that were managed successfully with transvaginal surgical excision.

## Methods

This is a case-series of three patients who have been referred with vaginal cysts mimicking cystocele in an urogynecology unit of a tertiary hospital. Ethical committee approval for reporting the case series was not required as routinely collected data were analyzed. However, fully informed consent was obtained from all the patients to report anonymized data and to use any photographic material. The inclusion criteria were: a) bothersome bulge as the primary symptom; b) vaginal cyst revealed after the clinical examination; c) Greek-speaker. The exclusion criteria were: a) history of pelvic prolapse surgery; b) primary diagnosis as a vaginal lesion. Demographic details and medical, surgical, and social history were obtained by the medical files and the out-patient notes. All women had: i) a detailed urogynaecological history taken; ii) a standard urogynaecological examination; iii) a trans-perineal 2D/3D-Pelvic Floor Ultrasound (PFUS) (Voluson S6, S10, General Electric); iv) response to specific conditions standardized questionnaires (ICIQ-UI SF, ICIQ-VS and PGI-I, and PGI-S). All patients underwent surgical removal of the cyst. The details of the surgical interventions and pathology reports were taken from the electronic files of the patients at the hospital´s database system. Patients were followed up at 6 weeks, at 6 months after the surgery. All available data were recorded on Microsoft Office Excel program. Means and standard deviations were calculated. Due to the small number of cases in the analysis, no statistical comparisons were performed.

**The surgical technique:** the excision took place in the operation room under regional anesthesia. Vaginal retractors were used for adequate exposure of the cyst. The vaginal epithelium over and around the lesions was infiltrated with normal saline using a thin 22-gauge needle. A scalpel was used to make an elliptical superficial incision to the vaginal epithelium overlying the cyst. Circumferential dissection of the cyst from the vaginal wall and the underlining urinary bladder was carried out sharply and bluntly using Metzenbaum scissors, knife or electrocautery as needed, starting from the upper and advancing to the lower edges of the cyst. Whenever the cyst has accidentally opened a figure of eight stitches (Coated Vicryl 2.0) was placed to prevent the spillage of the contents into the surgical field. The cyst was completely removed, and the edges of the vaginal defect were closed in two layers: at the deep, several interrupted coated vicryl 1.0 sutures were placed, whereas the overlying vaginal epithelium was sutured again with interrupted coated vicryl 1.0. Perioperative antibiotics and simple analgesics were administrated.

## Results

**The findings of the series:** the mean age, parity, and length of symptoms were 37±8.5 years, 1.7±1.5 children, and 19±15.1 months, respectively. All three women presented complaining of a vaginal bulge and they were referred for a cystocele for further management. The physical examination revealed a large, non-tender, mobile lesion on the anterolateral right vaginal wall. Due to the unusual mostly lateral presentation of the 'prolapsing' vaginal wall, an ultrasound examination was performed to elucidate the anatomical relationships of the prolapse. A trans-perineal 2D/3D PFUS was performed, showing a well-defined, round, thick-walled unilocular cystic lesion containing sub-echogenic contents without a septum, originating at the right lateral vaginal wall, extending from the introitus to the cervix of the uterus. Sonographic measurements ranged from 5.1 to 6.7 cm (maximum diameter). Pelvic floor ultrasound indicated clear borders of the cysts from the urethra and the urinary bladder, whereas no sign of communication between the cysts and the urinary tract was found. The patients were advised for surgical excision of the lesions. All patients were fully consented to and chose a total surgical excision of the lesion and were followed in the outpatient department. At 6 weeks and 6 months follow-up, all patients remained asymptomatic, with no sign of local erosion, and no further complaints reported. Patient satisfaction at 6 months follow-up with PGI-I score 1 was found in all patients. The patients were pleased with the results and would recommend the procedure to family or friends with similar conditions (Table 1)

**Table 1:** clinical characteristics of the patients of the study

Clinical feature	Case #1	Case #2	Case #3	Mean±SD
Age (years)	45	38	28	37±8.5
Parity	0	2	3	1.7±1.5
Symptoms' duration (months)	7	36	14	19±15.1
Primary symptom	Bothersome bulging prolapse	Bothersome bulging prolapse	Bothersome bulging prolapse	-
Other symptoms	No	Dyspareunia	Mild dyspareunia	-
Pre-operative POP-Q Aa	+2	+1	+2	1.7±0.6
Pre-operative POP-Q Ba	+3	+2	+2	2.3±0.6
PFUS max diameter (cm)	5.3	6.7	5.1	5.7±0.9
Histology	Gartner's cyst	Gartner's cyst	Müllerian cyst	-
Follow-up (6/52) and (6/52)	Asymptomatic	Asymptomatic	Asymptomatic	-
Post-operative POP-Q point Aa	-2	-2	-2	-2±0
Post-operative POP-Q point Ba	-2	-2	-2	-2±0
PGI-I	1	1	1	1±0

SD: standard deviation; POP-A: pelvic organ prolapse qualification system; PFUS: pelvic floor ultrasound; PGI-I: patient global impression of improvement

**Figure 1:**
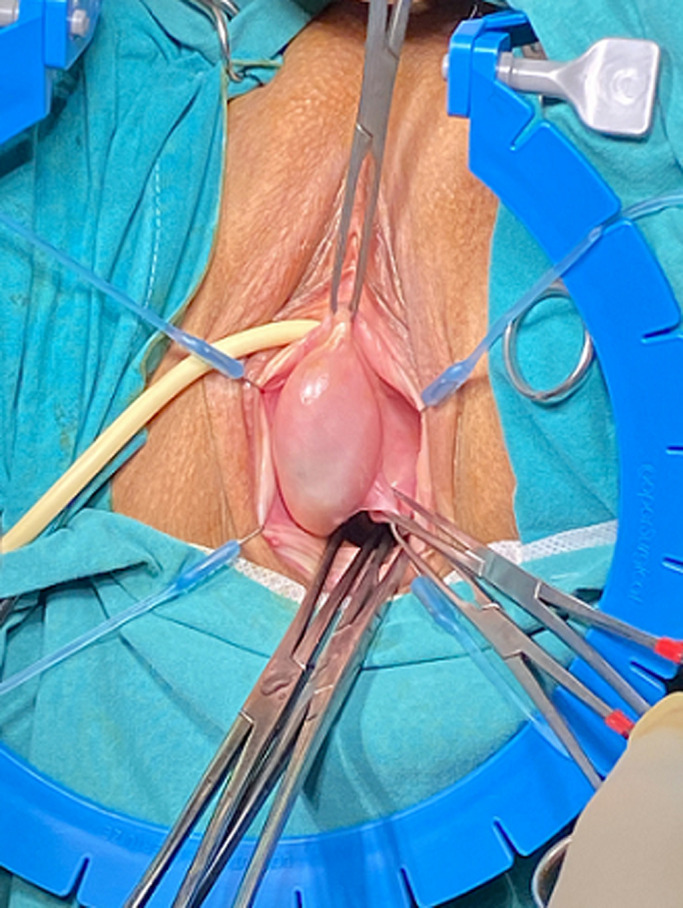
pre-operative image of the vaginal cyst mimicking prolapse patient #1

**Case 1:** a 45-year-old nulliparous woman presented with a feeling of fullness in her vagina for the last 6 months. The clinical examination showed a 4.3 x 5.3 x 3.2cm soft, non-tender cystic lesion on the upper right lateral vaginal wall (Figure 1). She reported no other symptoms ultrasound imaging was performed to detect any renal or other surrounding anomalies with which vaginal cysts may be associated. Surgical excision was decided from the patient because she felt really upset from her symptoms, and the histological examination revealed a low cuboidal no mucinous epithelium, advocating for a Gartner´s cyst. Six months following treatment the patient remained asymptomatic.

**Figure 2:**
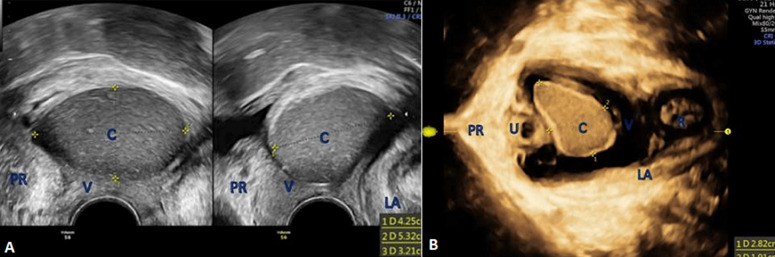
pelvic floor ultrasound (PFUS) evaluation of the cystic mass; a well-defined, round, thick-walled unilocular cystic lesion containing sub-echogenic contents without a septum, originating at the right lateral vaginal wall, extending from the introitus to the cervix of the uterus: A) 2D PFUS from patient #2; B) 3D PFUS - axial plane from patient #3; U=urethra, LA=levator ani, V=vagina, R=rectum, C=cyst, PR=pubic rami

**Case 2:** a 38-year-old G2P1 (one vaginal birth) female presented with a three years´ history of a heavy sensation of the bulge in the vagina associated with sexual problems and dyspareunia. The routine gynecological examination revealed a tumefaction at the anterolateral wall that her physician thought was a cystocele. Our speculum examination revealed a cystic lesion, painless, mobile with a soft consistency. Transperineal sonography revealed a large (2.9 × 2.1 × 6.7cm) vaginal cyst within the anterior lateral vaginal wall (Figure 2A). The patient consented for a surgical excision as treatment. The histologic evaluation was consistent with a Gartner´s cyst (low cylindrical - cuboidal cells). At 6 weeks and 6 months follow-up, the patient was asymptomatic and remains till today.

**Figure 3:**
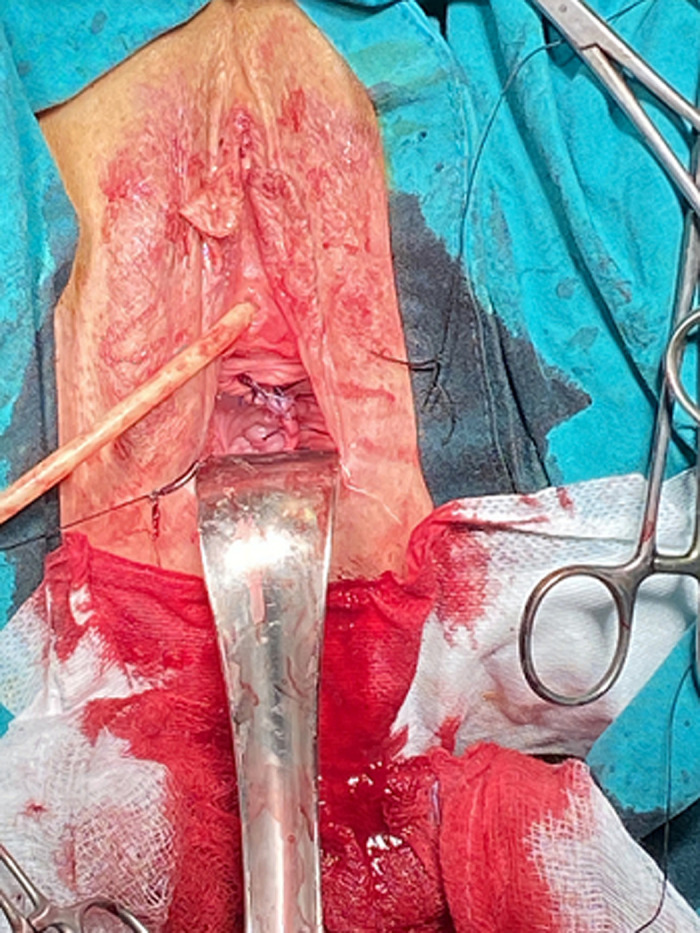
post-operative image after the surgical excision patient #3

**Case 3:** a 28-year-old G3P3 (three vaginal births) woman, was referred to our urogynecology unit because of feeling a bulge on her vagina. The bulge has been known for approximately one year, accompanied by mild dyspareunia. A pelvic examination revealed a 3.8 x 2.8 x 5.1cm non-tender, painless with soft consistency cystic lesion on her upper right anterior vaginal wall. During the preoperative assessment, the integrity of the urethra and bladder was evaluated easily with 3D PFUS (Figure 2B). An MRI was also given with the potential diagnosis of a hemorrhagic Gartner´s cyst. Surgical excision was performed with a Müllerian cyst as the histologic diagnosis (consisted of tall mucinous - columnar epithelium). The postoperative image is shown in the Figure 3. She remained with no symptoms at the 6 weeks follow-up.

## Discussion

Vaginal cysts are usually solitary, small, and asymptomatic; however, they can increase in size and provoke mild discomfort, fullness, dyspareunia to lower urinary tract symptoms. They can easily mimic other pathologies and be misdiagnosed as a cystocele. Currently, ultrasound examination is the first line investigation that permits accurate assessment and helps with the differential diagnosis of the various types of vaginal cysts and their relations with surrounding tissues and organs diagnosis and confirms the variety of co-existing anomalies. MRI can be used for the description of the borders of the lesion and the relationship to the adjacent organs. Retrograde urography into the cystic lesion permits the investigation of the proximity of the urinary tract to the cyst.

Full surgical excision constitutes a good management option. The vaginal approach we adopted to excise the cysts appears to have many advantages. First, in case that the complete resection of the cyst turns out to be impossible, marsupialization of the cyst into the vagina appears to be a viable and effective alternative [10]. Second, early mobilization and minimal invasiveness characterize the resection of these cysts by the vaginal route. Third, we successfully used the transabdominal ultrasound probe to intra-operatively check for the complete excision of the lesion and the proximity to other pelvic organs (i.e. the bladder and the ovaries). Vaginal cysts usually do not necessitate medical intervention as they may not cause symptoms. Kalva et al. had followed-up a 4-years-old girl with a Gartner´s duct cyst for 4 years; no indication for operative management appeared during that time [11]. The management of the symptomatic vaginal cysts is primarily surgical. A small number of authors have described their experience with patients complicated with large Gartner´s duct cyst during pregnancy. They preferred to drain the cyst the latest before the onset of labour without any complications [10]. Many authors have proposed marsupialization of the cyst, and it seems that it is a safe, minimally invasive technique with satisfactory long-term results [10]. The size of the cyst may cause difficulties during its excision, and a combined approach (abdominal-perineal or laparoscopic-perineal) may be adopted by the surgeons in order to secure its complete removal. In our case, this was feasible because the cystic wall was thick and permitted the dissection from the neighbouring organs at a clear surgical plane and with safety. Putting a drainage and packing the vagina at the end of the procedure is considered as safe practice in vaginal surgery, although these are interventions not confirmed as evidence-based practice.

## Conclusion

Large symptomatic vaginal cysts can present as genital prolapse and may be difficult to diagnose. Our case series prove that vaginal cysts are sometimes difficult to diagnose. Physicians must perform a careful full clinical examination and pelvic floor ultrasound is considered an acceptable method for setting the diagnosis. The surgical excision of them has a favorable outcome for the patients without complications.

### What is known about this topic

- Vaginal cysts usually come from embryologic remnants and the majority are asymptomatic;
- In rare cases, a vaginal cyst can be malignant or communicate with another pelvic organ as an ectopic ureter;
- Surgery appears to be the definitive treatment for symptomatic lesions.

### What this study adds

- Vaginal cysts can mimic pelvic organ prolapse and can be easily misdiagnosed;
- Pelvic floor ultrasound can contribute to an accurate assessment of a vaginal cyst;
- Vaginal surgery is the preferable approach for treatment, independent of the size and the location of the lesion.

## References

[1] Eilber KS, Raz S (2003). Benign cystic lesions of the vagina: a literature review. J Urol.

[2] Pradhan S, Tobon H (1986). Vaginal cysts: a clinicopathological study of 41 cases. Int J Gynecol Pathol.

[3] Blaivas JG, Flisser AJ, Bleustein CB, Panagopoulos G (2004). Periurethral masses: etiology and diagnosis in a large series of women. Obstet Gynecol.

[4] Deppisch LM (1975). Cysts of the vagina: classification and clinical correlations. Obstet Gynecol.

[5] Montella JM (2005). Vaginal Mullerian cyst presenting as a cystocele. Obstet Gynecol.

[6] Cil AP, Basar MM, Kara SA, Atasoy P (2008). Diagnosis and management of vaginal Mullerian cyst in a virgin patient. Int Urogynecol J Pelvic Floor Dysfunct.

[7] Binsaleh S, Al-Assiri M, Jednak R, El-Sherbiny M (2007). Gartner duct cyst simplified treatment approach. Int Urol Nephrol.

[8] Rios SS, Pereira LC, Santos CB, Chen AC, Chen JR, de Fátima B Vogt M (2016). Conservative treatment and follow-up of vaginal Gartner's duct cysts: a case series. J Med Case Rep.

[9] Bats AS, Metzger U, Le Frere-Belda MA, Brisa M, Lecuru F (2009). Malignant transformation of Gartner cyst. Int J Gynecol Cancer.

[10] Boujenah J, Ssi-Yan-Kan G, Prevot S, Chalouhi GE, Deffieux X (2014). A vaginal Gartner duct cyst presenting as a cystocele during pregnancy. Eur J Obstet Gynecol Reprod Biol.

[11] Kalva SP, Rammurti S, Subbarao D, Chittibabu N, Murthy VS (2001). Small ureterocele-like Gartner's duct cyst associated with ipsilateral renal aplasia: a case report. Australas Radiol.

